# ATRT–SHH comprises three molecular subgroups with characteristic clinical and histopathological features and prognostic significance

**DOI:** 10.1007/s00401-022-02424-5

**Published:** 2022-04-30

**Authors:** Aniello Federico, Christian Thomas, Katarzyna Miskiewicz, Niklas Woltering, Francesca Zin, Karolina Nemes, Brigitte Bison, Pascal D. Johann, Debra Hawes, Susanne Bens, Uwe Kordes, Steffen Albrecht, Hildegard Dohmen, Peter Hauser, Kathy Keyvani, Frank K. H. van Landeghem, Eva Løbner Lund, David Scheie, Christian Mawrin, Camelia-Maria Monoranu, Benedicte Parm Ulhøi, Torsten Pietsch, Harald Reinhard, Markus J. Riemenschneider, Astrid Sehested, David Sumerauer, Reiner Siebert, Werner Paulus, Michael C. Frühwald, Marcel Kool, Martin Hasselblatt

**Affiliations:** 1 Hopp Children’s Cancer Center (KiTZ), Heidelberg, Germany; 2 Division of Paediatric Neurooncology, German Cancer Research Center (DKFZ) and German Cancer Consortium (DKTK), Heidelberg, Germany; 3 Institute of Neuropathology, University Hospital Münster, Pottkamp 2, 48149 Münster, Germany; 4 Pediatric and Adolescent Medicine, Swabian Childrens’ Cancer Center, University Childrens’ Hospital Medical Center Augsburg and EU-RHAB Registry, Augsburg, Germany; 5 Department of Pathology and Laboratory Medicine, Children’s Hospital Los Angeles, Los Angeles, CA USA; 6 Institute of Human Genetics, Ulm University and Ulm University Medical Center, Ulm, Germany; 7 Department of Pediatric Hematology and Oncology, University Medical Center, Hamburg-Eppendorf, Hamburg, Germany; 8 Department of Pathology, McGill University, Montreal, QC Canada; 9 Department of Neuropathology, University Giessen, Giessen, Germany; 10 Department of Pediatric Oncology, 2nd Department of Pediatrics, Semmelweis University, Budapest, Hungary; 11 Institute of Neuropathology, University of Duisburg-Essen, Essen, Germany; 12 Division of Anatomical Pathology, Neuropathology Specialty Group, Department of Laboratory Medicine and Pathology, University of Alberta, Edmonton, Canada; 13 Department of Pathology, Rigshospitalet, Copenhagen, Denmark; 14 Department of Neuropathology, University Magdeburg, Magdeburg, Germany; 15 Department of Neuropathology, Institute for Pathology, University of Würzburg, 97080 Würzburg, Germany; 16 Department of Pathology, Aarhus University Hospital, Aarhus, Denmark; 17 Department of Neuropathology, University of Bonn Medical Centre, Bonn, Germany; 18 Asklepios Kinderklinik Sankt Augustin, Sankt Augustin, Germany; 19 Department of Neuropathology, Regensburg University Hospital, Regensburg, Germany; 20 Department of Paediatrics and Adolescent Medicine, University of Copenhagen, Copenhagen, Denmark; 21 Department of Pediatric Hematology and Oncology, University Hospital Motol, Prague, Czech Republic; 22 Princess Máxima Center for Pediatric Oncology, Utrecht, The Netherlands

**Keywords:** Atypical teratoid/rhabdoid tumor, Sonic hedgehog, DNA methylation profiling, Gene expression, OLIG2, GFAP, ASCL1, Neuroradiology, Prognosis, Overall survival

## Abstract

Atypical teratoid/rhabdoid tumor (ATRT) is an aggressive central nervous system tumor characterized by loss of SMARCB1/INI1 protein expression and comprises three distinct molecular groups, ATRT–TYR, ATRT–MYC and ATRT–SHH. ATRT–SHH represents the largest molecular group and is heterogeneous with regard to age, tumor location and epigenetic profile. We, therefore, aimed to investigate if heterogeneity within ATRT–SHH might also have biological and clinical importance. Consensus clustering of DNA methylation profiles and confirmatory t-SNE analysis of 65 ATRT–SHH yielded three robust molecular subgroups, i.e., SHH-1A, SHH-1B and SHH-2. These subgroups differed by median age of onset (SHH-1A: 18 months, SHH-1B: 107 months, SHH-2: 13 months) and tumor location (SHH-1A: 88% supratentorial; SHH-1B: 85% supratentorial; SHH-2: 93% infratentorial, often extending to the pineal region). Subgroups showed comparable *SMARCB1* mutational profiles, but pathogenic/likely pathogenic *SMARCB1* germline variants were over-represented in SHH-2 (63%) as compared to SHH-1A (20%) and SHH-1B (0%). Protein expression of proneural marker ASCL1 (enriched in SHH-1B) and glial markers OLIG2 and GFAP (absent in SHH-2) as well as global mRNA expression patterns differed, but all subgroups were characterized by overexpression of SHH as well as Notch pathway members. In a *Drosophila* model, knockdown of *Snr1* (the fly homologue of *SMARCB1*) in hedgehog activated cells not only altered hedgehog signaling, but also caused aberrant Notch signaling and formation of tumor-like structures. Finally, on survival analysis, molecular subgroup and age of onset (but not ASCL1 staining status) were independently associated with overall survival, older patients (> 3 years) harboring SHH-1B experiencing relatively favorable outcome. In conclusion, ATRT–SHH comprises three subgroups characterized by SHH and Notch pathway activation, but divergent molecular and clinical features. Our data suggest that molecular subgrouping of ATRT–SHH has prognostic relevance and might aid to stratify patients within future clinical trials.

**Supplementary Information:**

The online version contains supplementary material available at 10.1007/s00401-022-02424-5.

## Introduction

Atypical teratoid/rhabdoid tumor (ATRT) is a highly malignant central nervous system tumor characterized by loss of SMARCB1/INI1 protein expression [10]. ATRT comprises three molecular groups, i.e., ATRT–SHH, ATRT–TYR and ATRT–MYC [16]. ATRT–SHH represents the largest molecular group [11] and overexpression of members of the sonic hedgehog (SHH) and Notch signaling pathway are a characteristic feature [20, 40]. Protein expression of proneural marker Achaete-scute homolog 1 (ASCL1, also known as MASH1), a transcription factor [1] interacting with Notch signaling, has been proposed as a surrogate diagnostic marker for ATRT–SHH and has also been associated with improved outcome in ATRTs [40]. However, as not all ATRT–SHH express ASCL1, it remains uncertain whether ATRT–SHH patients in general experience better outcome [16]. Results from clinical trials and registries are conflicting: in the EU-RHAB registry, outcome of children harboring ATRT–SHH and ATRT–MYC was inferior to ATRT–TYR [11], while the Children’s Oncology Group Trial ACNS0333 reported a longer event-free survival for children harboring ATRT–SHH [34]. In contrast to ATRT–TYR, which are mainly of infratentorial location, ATRT–SHH may occur supratentorially and infratentorially, some tumors also affecting both compartments [28]. We and others have previously noted that ATRT–SHH exhibits further epigenetic heterogeneity, segregating into molecular subgroups associated with supratentorial or infratentorial location [16, 20]. We, therefore, aimed to investigate if epigenetic heterogeneity of ATRT–SHH is solely related to tumor location or might also have biological and clinical importance. Here, we demonstrate that ATRT–SHH comprises three robust molecular subgroups, which show characteristic clinical, histopathological and molecular features.

## Materials and methods

### Patient samples

Formalin-fixed paraffin-embedded samples of 65 ATRT–SHH were examined. Of those, 55 had been obtained in the context of the European Rhabdoid Registry EU-RHAB, and 10 were retrieved from the archives of the Institute of Neuropathology Münster. EU-RHAB and the tumor bank of the Institute of Neuropathology Münster have received continuous local ethics committee approval (Ethics committee of the University Hospital Münster) and patients or the guardians provided informed consent for scientific use of archival materials.

### Neuroimaging

Information on tumor location was retrieved from patient records. Furthermore, preoperative magnetic resonance imaging data of 42/65 patients was available for neuroradiological review. Maximal diameter of the tumor was determined, and tumor volume was calculated (approximation formula: a × b × c × 0.5). Furthermore, for each case, the maximal tumor area in the sagittal plane was determined and projected on a schematic drawing of the CNS according to molecular subgroup. Extent of resection was assessed by reviewing patient records and postoperative imaging studies.

### Immunohistochemistry

Immunohistochemistry for ASCL1 was performed using a mouse monoclonal antibody (BD Bioscience, #556604, 1:100, high pH antigen retrieval) on a Bond RXm (Leica Biosystems) or a DAKO Link48 (Agilent) automated staining system at two different institutions (Children's Hospital Los Angeles and University Hospital Münster). Samples stained on both platforms in parallel for validation yielded comparable results. Immunohistochemical staining for GFAP (#GA524, Agilent), OLIG2 (#18953, Immuno-Biological Laboratories, Inc.), and synaptophysin (#M7315, Agilent), was performed using the streptavidin–biotin method on an automated staining system (DAKO OMNIS, Agilent). For the purpose of the present study, immunohistochemical staining results were rated as absent, focal (< 5% of tumor cells) and present (≥ 5% of tumor cells).

### Molecular genetic examinations

Fluorescence *in situ* hybridization (FISH) of the *SMARCB1* locus, *SMARCB1* sequencing and multiplex ligation-dependent probe amplification (MLPA) using the SALSA MLPA P258 (SMARCB1) kit (MRC-Holland) were performed as described previously [15, 22].

### DNA Methylation profiling

After DNA isolation from formalin-fixed paraffin-embedded tumor samples, purification and bisulfite conversion using standard protocols provided by the manufacturer, all samples were analyzed using the HumanMethylation450 BeadChip array or the MethylationEPIC BeadChip array (Illumina Inc., San Diego, CA). Raw IDAT files from both array types were loaded into the R environment (version 4.0.1) using the minfi package (version 1.34). CpG sites represented on the MethylationEPIC BeadChip array, but not on the HumanMethylation450 BeadChip array were excluded from analysis. In addition, the following filtering criteria were applied: removal of probes targeting the X and Y chromosomes, removal of probes containing a single nucleotide polymorphism (dbSNP132 Common) within five base pairs of and including the targeted CpG site, and probes not mapping uniquely to the human reference genome (hg19) allowing for one mismatch. A total of 384,232 probes were kept for downstream analyses. Copy-number variation analysis from DNA methylation array data was performed using the conumee Bioconductor package and chromosomal gains and losses were examined by manual inspection of each profile. CNV plots were visualized using IGV (version 2.11.4; Broad Institute).

### DNA methylation class prediction and t-SNE analysis

To confirm the tumor identity of the 65 tumor samples as ATRT–SHH, we referred to the class prediction scores generated using the Brain Tumor Classifier version 12.3 and compared their DNA methylation profiles to the CNS tumor DNA methylation reference cohort of the Molecular Neuropathology (MNP, www.molecularneuropathology.org) platform. This source includes a large and constantly expanding collection of DNA methylation data covering the great majority of the currently known CNS tumor classes and subclasses. This classification method provided by this tool, based on a random forest algorithm and selection on the most informative DNA methylation probes [2], assigned a classification score (ranging from 0 to 1) to each diagnostic case as estimation of similarity to any of the CNS tumor classes (and/or subclasses) represented in the reference. For output interpretation, we considered all the calibrated classifier scores with a cutoff ≥ 0.9 as optimal for a valid prediction.

Next, we aimed at visualizing the distribution of our cohort’s cases based on their DNA methylation profiles and the eventual formation of multiple independent sub-clusters. We projected the 65 DNA methylation data into a large (> 83.000) DNA methylation data set, including the MNP references cohort cases, DNA methylation tumor samples generated by MNP’s involved parties (University Hospital Heidelberg, Germany; German Cancer Research Center, Germany; German Consortium for Translational Cancer Research, Germany) [3] and all the DNA methylation data uploaded on MNP website (www.molecularneuropathology.org). We will herein refer to this data set as “Heidelberg DNA methylation data set”. Data projection was computed using a t-distributed stochastic neighbor embedding (t-SNE) dimensionality reduction algorithm (Rtsne package), using as input a beta-value matrix of the top 1000–5000 differentially methylated probes.

### Consensus clustering

Consensus clustering was performed on the matrix of beta values using the R/bioconductor package cola (version 2.0.0) [12]. Various combinations of feature selection and partitioning methods were adopted to fit consensus clustering models with k subgroups ranging from 2 to 6. Standard deviation (SD), coefficient of variance (CV), median absolute deviation (MAD) and ability to correlate to other rows (ATC) were used as feature selection methods. The following partitioning methods were used to separate samples into subgroups ranging from 2 to 6 classes: hierarchical clustering with cutree (hclust), k-means clustering (kmeans), spherical k-means clustering (skmeans), partitioning around medoids (pam) and model-based clustering (mclust). Similar to the evaluation of a large DNA methylation array data set in [12], setting SD as the feature selection method resulted in the most distinctive DNA methylation profile observed on simple clustering heatmap (Supplementary Figure S1). Therefore, partitioning methods were evaluated in combination with SD as the feature selection method. The models were assessed to determine the optimal fit using the mean silhouette score, the 1-proportion of ambiguous clustering (PAC) score, concordance, and the Jaccard index. In addition, consensus heatmaps and membership heatmaps (illustrating the membership of every individual partition generated from random subsets of the original matrix) were visually inspected.

### Gene expression profiling

Due to the lack of RNA material for the 65 cohort samples, we performed a transcriptomic analysis of additional 22 ATRT–SHH cases, for which RNA data (Affymetrix Human Genome U133 Plus 2.0 array) were available, and that based on our DNA methylation sub-clustering analysis of the extended cohort (*n* = 87) could be categorized as one of the three SHH subgroups (SHH-1A *n* = 10, SHH-1B *n* = 7, and SHH-2 n = 5). Transcriptome data of ATRT–TYR (*n* = 21) and ATRT–MYC (*n* = 7) were also included for comparative analyses among ATRT subgroups. Data analysis was performed in R2 (https://r2.amc.nl). Normalized (MAS5.0) and Log-2 transformed data was used as input for differentially gene expression (DE) analysis (*T* test applied for two group comparison, ANOVA test for multiple group comparisons). Top DE genes, based on their fold change (FC; > 1) and *p* value cutoff (0.05), were selected for heatmap visualization (ComplexHeatmap R package, version 2.5.5). A full list of DE genes is shown in Supplementary Table 2. Gene ontology of the DE genes has been performed using the DAVID web tool (https://david.ncifcrf.gov/). We tested brain region gene specificity using The Human Protein Atlas (https://www.proteinatlas.org/) and Allen Human Brain Atlas (https://human.brain-map.org/) as sources.

### Drosophila experiments

Flies were raised on standard cornmeal–yeast–agar medium. For *Snr1* knock-down, UAS-Snr1RNAi (P{KK101602}VIE-260B #v108599 VDSC) line and controls: UAS-mCherryRNAi (y[1] sc[*] v[1] sev[21]; P{y[+ t7.7] v[+ t1.8] = VALIUM20-mCherry}attP2, #35785 BDSC) or w1118 (#3605 BDSC) were crossed with lines: en-gal4 (P{en2.4-Gal4}e16E, FlyBase ID FBrf0098595), UAS-Dcr, en-gal4 UAS-GFP [P{w[+ mC] = UAS-Dcr-2.D}1, w[1118]; P{w[+ mW.hs] = en2.4-GAL4}e16E, P{w[+ mC] = UAS-2xEGFP}AH2, #25752 BDSC] or en-gal4 UAS-RFP NRE-GFP (w[1118]; P{w[+ mW.hs] = en2.4-GAL4}e16E, P{w[+ mC] = UAS-myr-mRFP}1, P{w[+ m*] = NRE-EGFP.S}5A, #30729 BDSC), respectively. For condition 25 °C: flies were raised at 25 °C. For condition 29 °C: F0 and F1 till 2nd instar larva were kept at 18 °C, then larvae were transferred to 29 °C for 60 h before dissections. Larvae were dissected as wandering 3rd instar (L3) when larval growth is accomplished. RNAi expression together with over expression of Dcr and higher temperatures increased frequency of phenotype: growth alteration, confirming its specificity to RNAi. Frequencies were 31% (8/26, 25 °C), 45% (19/42, 25 oC + Dcr) and 50% (5/10, 29 °C + Dcr), respectively. For fluorescence microscopy, halves of L3 larvae or “open-stretched” L3 were fixed in 3.7% pFA/PBS for 20 min, permeabilized by 0.15% Triton X-100/PBS for 1 h and embedded in medium containing DAPI (Roth) at least 1 h prior to imaging. Image stacks were acquired with an LSM 700 confocal microscope (Zeiss), using a 10X Plan Apo ([NA] = 0.45) objective lens. Images are shown as *z* maximal projections of stacks. Tissues were analyzed at single optical sections for abnormalities. The volume of brain lobes was calculated as described previously [19] using Image J software.

### Statistics

Differences between the three molecular subgroups were examined by Kruskal–Wallis ANOVA or Chi-Square test. Survival analysis was performed using Kaplan–Meier curves and the Log-Rank test. For multivariate analysis, Cox Regression was performed using a backward Wald approach. All statistical analyses were done using the SPSS software package (Version 28.0.1.0, IBM).

## Results

### ATRT–SHH represents a group with heterogeneous clinical features

The series comprised 62 children and three young adults harboring ATRT–SHH. The age of the 33 female and 32 male patients ranged from 0 to 28 years (median: 1 year). Thirty-five tumors (54%) were of supratentorial location and 12 (18%) were located infratentorially, while 18 tumors (28%) exhibited an infratentorial and a supratentorial component. Of note, the latter group mainly comprised infratentorial tumors extending to midbrain structures and the pineal region (16/18). Two patients harbored independent infratentorial and supratentorial lesions, while in two patients two independent supratentorial lesions were encountered. Median tumor volume was 44 cm^3^. Median tumor volumes of supratentorial tumors (48 cm^3^) and tumors exhibiting an infratentorial and a supratentorial component (52 cm^3^) did not differ significantly from that of infratentorial tumors (24 cm^3^, *P* = 0.07 Kruskal–Wallis ANOVA). Gross total resection was achieved in 24/56 cases for which information on extent of resection was available (43%) and the proportion of tumors, in which gross total resection was achieved, did not differ significantly by tumor location.

Histopathologically, all tumors were diagnosed as ATRT according to current WHO criteria. In line with previous observations [46], poorly differentiated small round and blue-celled areas prevailed in the majority of tumors and some samples contained only few rhabdoid tumor cells. Of note, some cases (especially in older patients) also showed a glial appearing tumor component, which in two cases initially had resulted in misinterpretation as malignant glioma. All tumors, however, showed complete loss of nuclear SMARCB1/INI1 protein expression and were unequivocally classified as ATRT–SHH using the Heidelberg Brain Tumor Classifier [median calibrated classifier score: 1.00 (Classifier version 12.3)]. Genetic alterations affecting the *SMARCB1* locus comprised complete loss of 22q (*n* = 24), *SMARCB1* deletions (*n* = 30) as well as *SMARCB1* point mutations (*n* = 30). Point mutations often affected both alleles (*n* = 14) and exon 2 mutations were the most frequent (*n* = 12; for more details see Supplementary Table 1).

### ATRT–SHH comprises three robust epigenetic subgroups

**Fig. 1:**
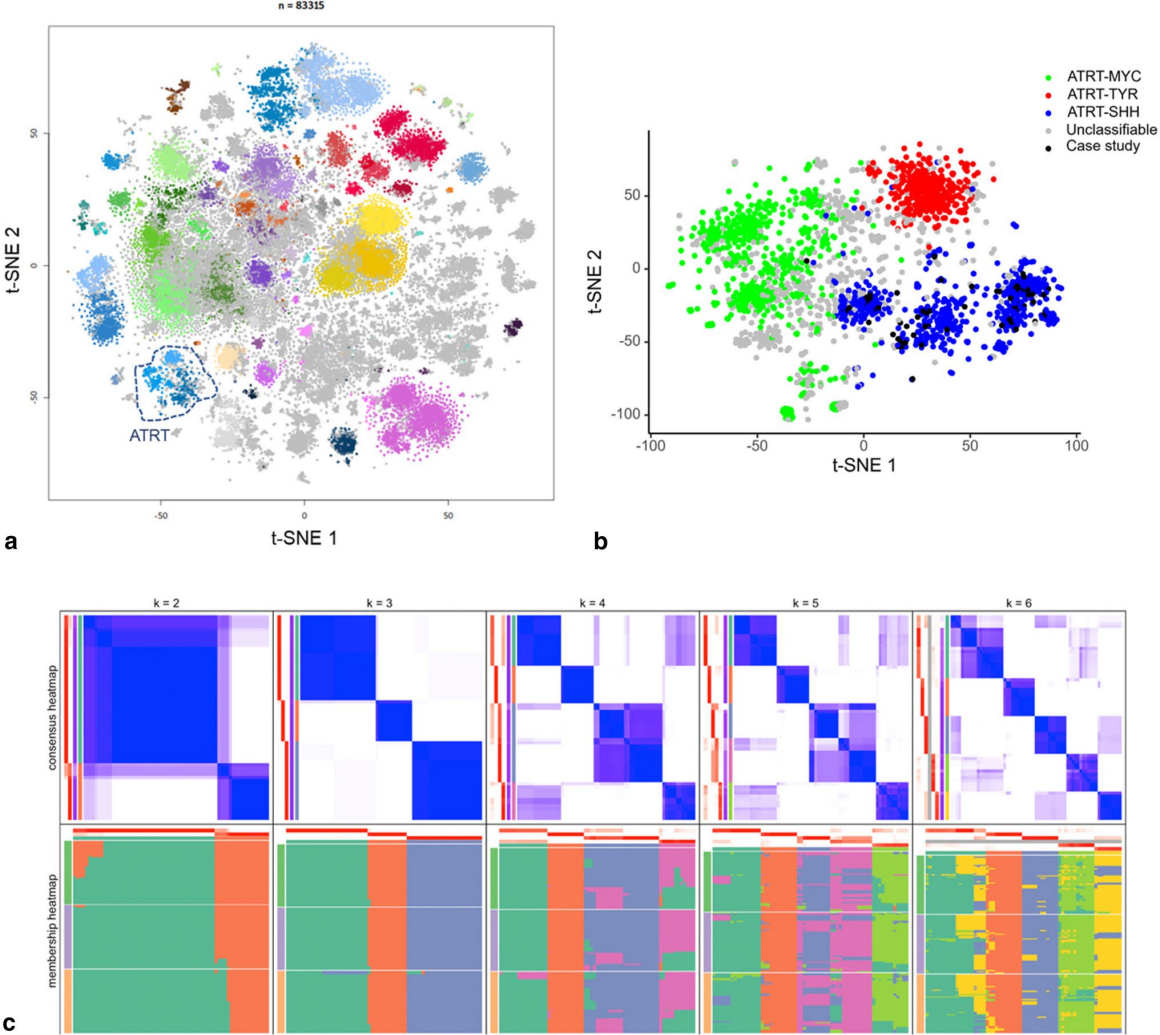
DNA methylation profiling of ATRT–SHH reveals three distinct molecular subgroups. Unsupervised t-SNE analysis of the tumor cases together with > 83.000 samples of the molecularneuropathology.org data set (**a**) segregates ATRT–SHH into three DNA methylation subgroups. A more focused analysis of the ATRT cases within the Heidelberg data set (*n* = 1919) further suggests the presence of three ATRT–SHH subgroups (**b**). Black dots indicate the cases of this study. Unsupervised clustering analysis using spherical k-means clustering for *k* = 2–6 revealed most stable consensus heatmaps and membership partitioning for *k* = 3 clusters (**c**)

We next visualized the DNA methylation-based clustering behavior of our study cohort cases within the large and comprehensive Heidelberg DNA methylation data set. On the t-SNE projection, the cohort tumors are all clustered within the main ATRT group (Fig. 1a). To gain a more in-depth focus on ATRT tumor subclasses, we performed a subcluster DNA methylation analysis including our 65 diagnostic cases alone or together with additional tumor data forming the main ATRT cluster observed in the overall t-SNE projection of the Heidelberg DNA methylation data sets. This included confirmed (classifier score ≥ 0.9) cases from each of the known ATRT molecular groups (ATRT–SHH, ATRT–TYR, and ATRT–MYC) and unconfirmed /unclassifiable (< 0.9) data (total *n* = 1919). T-SNE projection of this set allowed us to observe that all 65 tumor cases co-clustered along with the other ATRT–SHHs and, strikingly, they were distributed across three separated substructures (Fig. 1b), suggesting the possibility of three independent ATRT–SHH epigenetic subgroups. For optimal selection of stable subgroups, consensus clustering of the 65 ATRT–SHH was performed on the matrix of beta values using the cola framework [12] with standard deviation as the feature selection method for the top 1000, 2000, and 4000 most variable probes, respectively. Various clustering methods were applied, including hierarchical clustering, k-means clustering, spherical k-means clustering, partitioning around medoids, and model-based clustering. Inspection of the consensus heatmaps revealed most stable partitioning for *k* = 3 clusters using k-means clustering method (Fig. 1c, Supplementary Figure S2). Stability metrics for the combination of SD and k-means clustering further support *k* = 3 as the optimal number of clusters (1-PAC = 1.00, mean silhouette score 0.98, concordance 0.99; Supplementary Figure S3). Based on these observations, we chose to designate these three epigenetic subgroups as SHH-1A (*n* = 25), SHH-1B (*n* = 13), representing mainly supratentorial tumors, and SHH-2 (*n* = 27), mainly representing tumors with predominant infratentorial location.

### ATRT–SHH subgroups differ by age distribution and tumor location

**Fig. 2:**
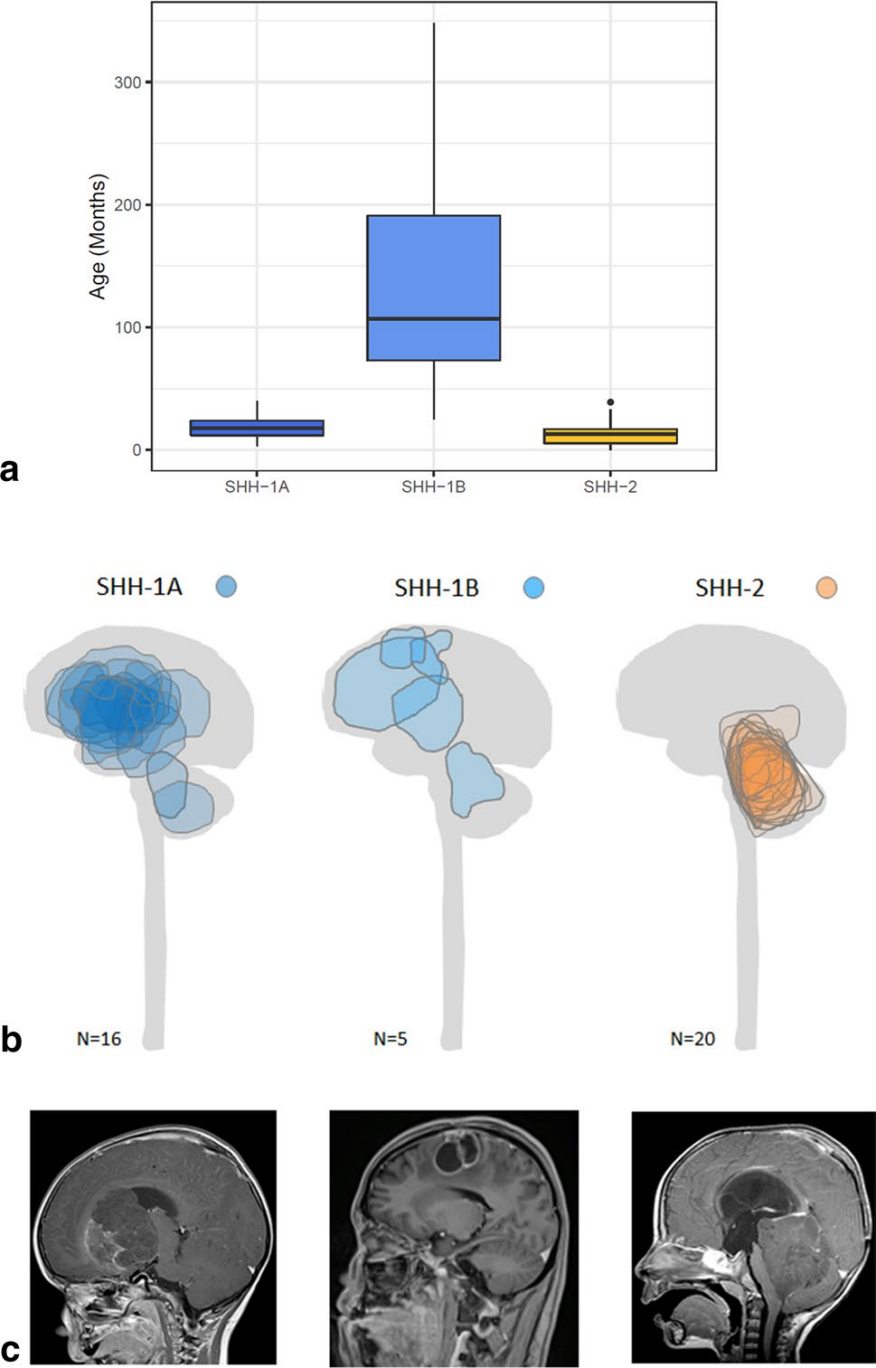
Age distribution and tumor location of ATRT–SHH subgroups. Age distribution of ATRT–SHH subgroups (**a**). Note that median age of onset of patients harboring SHH-1B tumors is significantly higher as compared to SHH-1A and SHH-2 tumors (Kruskal–Wallis ANOVA *p* < 0.0001). Tumor location of SHH-1A and SHH-1B tumors is mainly supratentorial, whereas the majority of SHH-2 tumors represents infratentorial tumors, often extending to midbrain structures and the pineal region (**b**). For visualization, maximal tumor areas in the sagittal plane were determined and projected on a schematic drawing of the CNS. Representative magnetic resonance images are also given (**c**)

The three ATRT–SHH subgroups markedly differed regarding age at diagnosis. The median age at diagnosis of patients harboring SHH-1B was 107 months (range: 25–347 months), whereas median age at diagnosis of patients harboring SHH-1A [18 months (range: 3–40 months)] and SHH-2 [13 months (range: 0–39 months)] was significantly lower (Kruskal–Wallis-ANOVA *p* < 0.0001; Fig. 2a). As mentioned above, SHH-1A (22/25; 88%) and SHH-1B (11/13; 85%) were mainly of supratentorial location. In contrast, only 2/27 (7%) SHH-2 were located supratentorially, whereas the majority of SHH-2 represented infratentorial tumors [8/27 (30%)] or infratentorial tumors extending to midbrain structures and the pineal region [17/27 (63%) Fig. 2b, c; Chi-Square 42.65; df:4; *p* < 0.00001]. Median tumor volumes (50 cm^3^ in SHH-1A and 51 cm^3^ in SHH-1B as compared to 35 cm^3^ in SHH-2) did not differ significantly and the proportion of tumors for which gross total resection could be achieved was comparable across subgroups (Chi-Square: 1.36; df: 2; n.s.).

### ATRT–SHH subgroups show comparable *SMARCB1* mutational profiles, but differences in the proportion of pathogenic/likely pathogenic *SMARCB1* germline variants

**Fig. 3:**
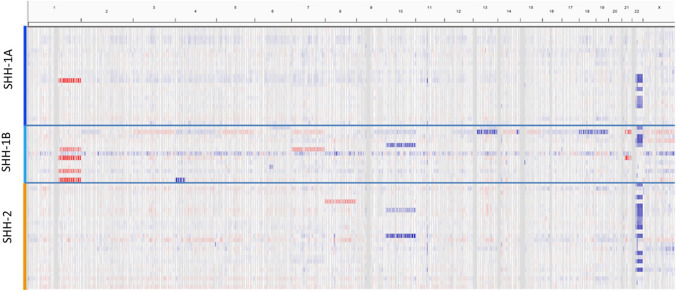
Copy-number variation (CNV) analysis of ATRT–SHH subgroups. CNV analysis indicates frequent loss of Chr22q across all three ATRT–SHH subgroups. Note further chromosomal alterations (including gains of chromosome 1q) are more frequent in SHH-1B

The proportion of cases showing heterozygous or homozygous losses of the *SMARCB1* locus as assessed by FISH was comparable across subgroups. Similarly, the proportion of cases showing *SMARCB1* point mutations detected by Sanger sequencing as well as their distribution across *SMARCB1* exons was comparable. On analysis of DNA methylation array intensity data, the proportion of cases showing complete losses of 22q were comparable in SHH-1A [5/25 (20%)], SHH-1B [3/13 (23%)] and SHH-2 [13/27 (48%); Chi-Square 5.336, df: 2; n.s.]. In addition to 22q losses, analysis of DNA methylation array intensity data yielded few further recurrent chromosomal alterations. However, the percentage of cases demonstrating further chromosomal alterations differed among molecular subgroups and accounted for 1/25 (4%) in SHH-1A, 4/27 (15%) in SHH-2 and 6/13 (46%) in SHH-1B (Chi-Square: 10.96; df: 2; *P* = 0.004). Recurrent copy number alterations involved gains of whole chromosome arm 1q in SHH-1A (1/25) and SHH-1B (4/13), loss of chromosome 10 in SHH-2 (2/27) and SHH-1B (1/13) as well as gains of chromosome 7 in SHH-1B (2/13; Fig. 3). Furthermore, the proportion of patients with pathogenic/likely pathogenic *SMARCB1* germline variants differed among subgroups. While pathogenic/likely pathogenic *SMARCB1* germline variants were only encountered in 4/20 (20%) patients harboring SHH-1A and were absent in patients harboring SHH-1B (0/6; 0%), they were present in 15/24 (63%) patients harboring SHH-2 (Chi-Square: 12.54; df: 2; *P* = 0.002).

### ATRT–SHH subgroups exhibit differential protein expression of proneural marker ASCL1 and glial markers OLIG2 and GFAP

**Fig. 4:**
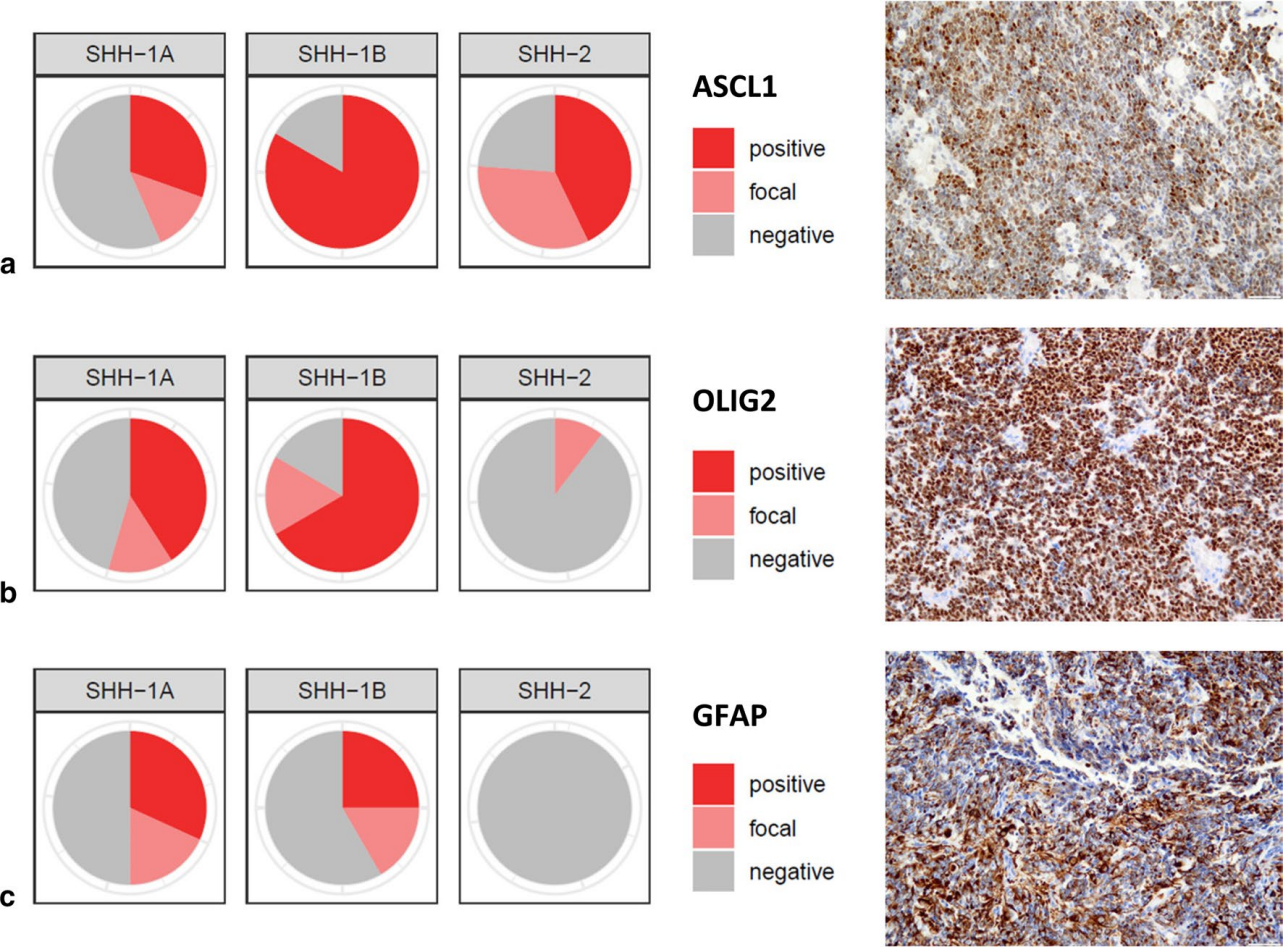
Immunohistochemical staining profile of ATRT–SHH subgroups. The three ATRT–SHH subgroups show differential protein expression of ASCL1 (**a**), OLIG2 (**b**) and GFAP (**c**). Polar plots and representative positive staining results are given. Scale bars denote 100 µm

We observed that the proportion of cases staining positive for ASCL1, OLIG2 and GFAP differed markedly among subgroups (Fig. 4). Whereas the vast majority of SHH-1B [10/12 (83%)] stained positive for ASCL1 (> 5% of tumor cells), positive ASCL1 staining was less frequent in SHH-1A [7/23 (30%) and SHH-2 [9/21 (43%); Chi-Square: 9.044; *P* = 0.01]. In contrast, many SHH-1A and SHH-1B cases stained positive for OLIG2 [9/22 (41%) and 8/12 (67%), respectively], whereas positive OLIG2 staining (> 5% of tumor cells) was not encountered in any SHH-2 [0/23 (0%); Chi-Square: 18.849; df: 2; *P* < 0.001]. Similarly, a proportion of SHH-1A and SHH-1B cases showed positive staining for GFAP [7/22 (32%) and 3/12 (25%), respectively], whereas positive GFAP staining (> 5% of tumor cells) was not encountered in any SHH-2 examined [0/18 (0%); Chi-Square: 6.787; df: 2; *P* = 0.034]. In summary, proneural marker ASCL1 was expressed across subgroups, but overrepresented in SHH-1B. In contrast, positive OLIG2 and GFAP staining (> 5% of tumor cells) was present in many SHH-1A and SHH-1B, but absent in SHH-2.

### ATRT–SHH subgroups exhibit differences in global gene expression profiles, but similar expression levels of SHH and Notch pathway members

**Fig. 5:**
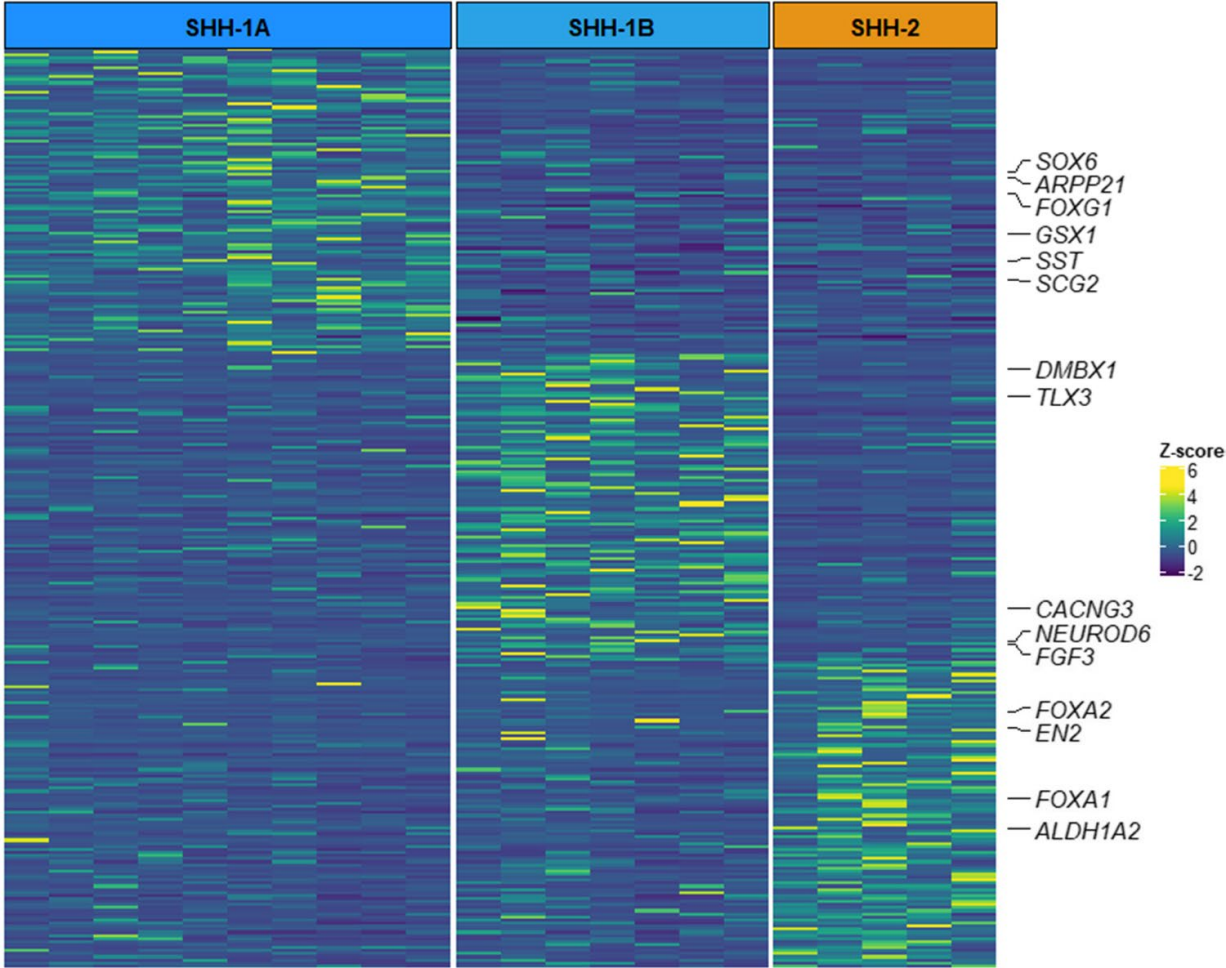
Gene expression profiling of ATRT–SHH subgroups. Differential expression analysis of ATRT–SHH subgroups showing gene sets specifically overexpressed in each subgroup. The heatmap displays the top 100 up-regulated genes based on their Log2 fold change. Gene intensities plotted in the heatmap are in *Z* score format. Brain-specific genes, according to the Human Protein Atlas and Allen Human Brain Atlas, are highlighted

We next investigated whether the identified ATRT–SHH subgroups also showed transcriptomic differences. Due to the lack of RNA availability in our cohort, we examined gene expression profiles of additional ATRT cases (*n* = 22) that could be categorized as SHH-1A (*n* = 10), SHH-1B (*n* = 7) or SHH-2 (*n* = 5) based on DNA methylation profiling data (Supplementary Figure S4 and Supplementary Table 2). On differential gene expression analysis to identify genes upregulated in each of the SHH subgroups as compared to the other two, we observed that the three SHH subgroups exhibited distinct transcriptional profiles with the upregulation of several brain-specific genes (Fig. 5 and Supplementary Table 2). Functional annotation analysis of subgroup-specific genes showed the enrichment of the GO term “Central Nervous System development” (GO: 0,007,417) for SHH-1A and SHH-1B subgroups, while in SHH-2 upregulated genes were linked to glutamatergic synaptic transmission (GO: 0,007,215, GO: 0,035,249), dopaminergic neuron differentiation (GO: 0,071,542) and hindbrain development (GO: 0,030,902) (Supplementary Table 2). SHH-1A upregulated genes included *GSX1* and *FOXG1*, predominantly expressed in the hypothalamus and cortex according to the Human Protein Atlas. FOXG1 is a known effector and interactor of the SHH pathway [8] and has been found to be deregulated in glioma [4]. Other SHH-1A associated genes were the neuroendocrine-specific tumor genes *SST* and *SCG2*; *ARPP21*, encoding an RNA-binding protein found in glioma [45], and *SOX6*, a tumor marker expressed by not fully differentiated neuronal and glial cells [41]. SHH-1B upregulated genes included neuronal progenitor cell markers *NEUROD6* and *TLX3* as well as other brain-specific genes (e.g., *CACNG3* and *DMBX1*). The SHH-2 gene signature comprised *ALDH1A2* and *EN2*, which are involved in the organization of the hindbrain [9, 21] and upregulated in ependymoma PFA2 tumors [29], as well as the dopaminergic neuron markers *FOXA1* and *FOXA2* [33]. Next, we also examined expression of genes coding for members of the SHH and Notch pathway known to be overexpressed in ATRT–SHH. Overall, these genes were overexpressed in each of the three ATRT–SHH subgroups (also in comparison to ATRT–TYR and ATRT–MYC) and no particular differences were observed between ATRT–SHH subgroups (Supplementary Figure S5).

### Knockdown of *Snr1* in hedgehog activated cells causes aberrant hedgehog and Notch signaling and formation of tumor-like structures in a fly model of SMARCB1 deficiency

**Fig. 6:**
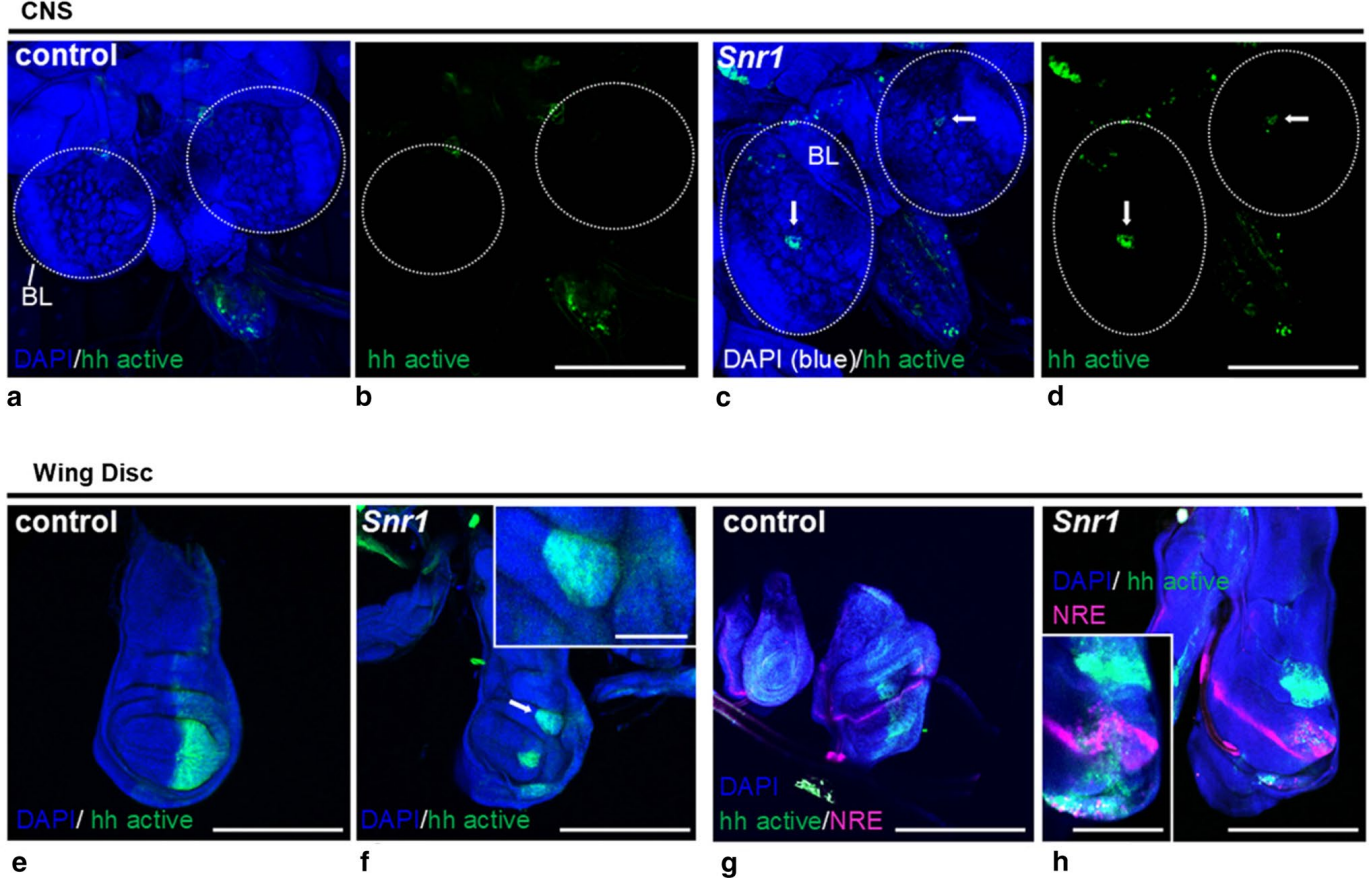
Knockdown of the *SMARCB1* homologue *Snr1* in hedgehog activated cells causes aberrant hedgehog as well as Notch signaling and formation of tumor-like structures in the fly model. In the larval central nervous system (**a**–**d**), *Snr1RNAi* enhances hedgehog signaling. In the representative images, eGFP (green) expressed with UAS promoter under control of *engrailed*-gal4, marks engrailed positive/hedgehog activated cells in controls (**a**, **b**; *n* = 12) and upon *Snr1RNAi* knockdown (**c**, **d**; *n* = 13). Note eGFP-positive cell clusters (arrows) in the brain lobes (BL) upon *Snr1* knockdown not visible in control animals. In the imaginal wing disc (**e**–**h**), *Snr1* knockdown causes growth defects and atypical *engrailed* expression pattern in the posterior compartments, visualized by eGFP (green) in controls (**e**; *n* = 16) and upon *Snr1RNAi* knockdown (**f**; *n* = 19). Note tumor-like structure (arrow and inset) upon *Snr1RNAi* knockdown. *Snr1* knockdown in hedgehog activated cells also results in ectopic Notch signaling, especially in the posterior compartment of the wing disc (**g**, **h**). Expression of *engrailed* is reported with mRFP (shown as green) under control of the UAS/GAL4 system while activation of Notch: ‘Notch-on’ state with GFP (shown in magenta) under control of NRE (Notch responding element), in controls (**g**; *n* = 9) and in *Snr1RNAi* (**h**; *n* = 10). Note characteristic stripe-like Notch expression pattern at the boundary of the dorso-ventral line in controls (**g**) that is more intense in the *engrailed*-negative anterior compartment of *SnrRNAi* wing discs (**h**). In the posterior compartment of *Snr1RNAi* wing discs, additional ‘Notch-on’ clusters (magenta) are visible, which are frequently associated with *engrailed* (green) and abnormal tumor-like structures (inset). Nuclei counterstained with DAPI (blue). Scale bars denote 200 µm or 100 µm (insets). Fly genotypes are described in detail in the Materials and methods section

Despite the observed heterogeneity of DNA methylation profiles and gene expression patterns among the three ATRT–SHH subgroups, all were characterized by overexpression of SHH and Notch pathway members. In ATRT–SHH, genetic alterations activating the SHH pathway are absent [16]. This suggests that regulatory mechanisms of SHH and/or Notch pathway may be affected by SMARCB1-deficiency and also raises the possibility of interactions between SHH and Notch signaling in the tumorigenesis of ATRT–SHH. We, therefore, employed a *Drosophila* model to explore whether SMARCB1-deficiency indeed causes aberrant hedgehog and Notch signaling. Knockdown of *Snr1*, the fly homologue of *SMARCB1*, in hedgehog activated cells was performed using the Gal4/UAS system (*UAS-Snr1RNAi*) under the control of the *engrailed* promotor, which is a hedgehog pathway target gene and forms a positive feedback loop for hedgehog expression [17]. The gross morphology of CNS structures of late 3rd instar larvae upon *Snr1* knockdown in *engrailed* cells was comparable to control and brain lobe volumes did not differ (*t*-test n.s.). However, in contrast to control flies, which showed none or only very weak GFP expression in the brain lobes (Fig. 6a, b), upon *Snr1* knockdown clusters of GFP-labeled *engrailed* expressing cells were visible within the brain lobes of all tested animals (Fig. 6c, d).

In the wing disc, a larval structure that represents a well characterized paradigm for the role of hedgehog signaling during development [14], normal morphology under control conditions (Fig. 6e) was strongly affected when *Snr1* was knocked down in *engrailed* expressing cells. Tumor-like accessory structures were encountered (Fig. 6f) and GFP-labeled *engrailed* expressing cells were enriched in such structures (Fig. 6f, inset). We also tested whether downregulation of *Snr1* in *engrailed* expressing cells affects Notch signaling activity by monitoring fluorescent Notch reporter NRE–GFP [36]. Whereas in controls the typical thin stripe-like Notch expression pattern at the boundary of the dorsoventral line was observed (Fig. 6g), upon *Snr1* knockdown in hedgehog activated cells the stripe-like Notch expression pattern was broader and additional clusters with ectopic Notch activation were encountered (Fig. 6h). These clusters were frequently associated with *engrailed* positive regions and abnormal tumor-like structures (Fig. 6h, inset). Taken together, *Snr1* knockdown in *engrailed* expressing cells causes aberrant hedgehog and Notch signaling and is associated with formation of tumor-like structures in the fly model, suggesting that SMARCB1-deficiency may have a similar role on SHH and Notch activation in human ATRT–SHH.

### Molecular subtyping of ATRT–SHH has clinical relevance

**Fig. 7:**
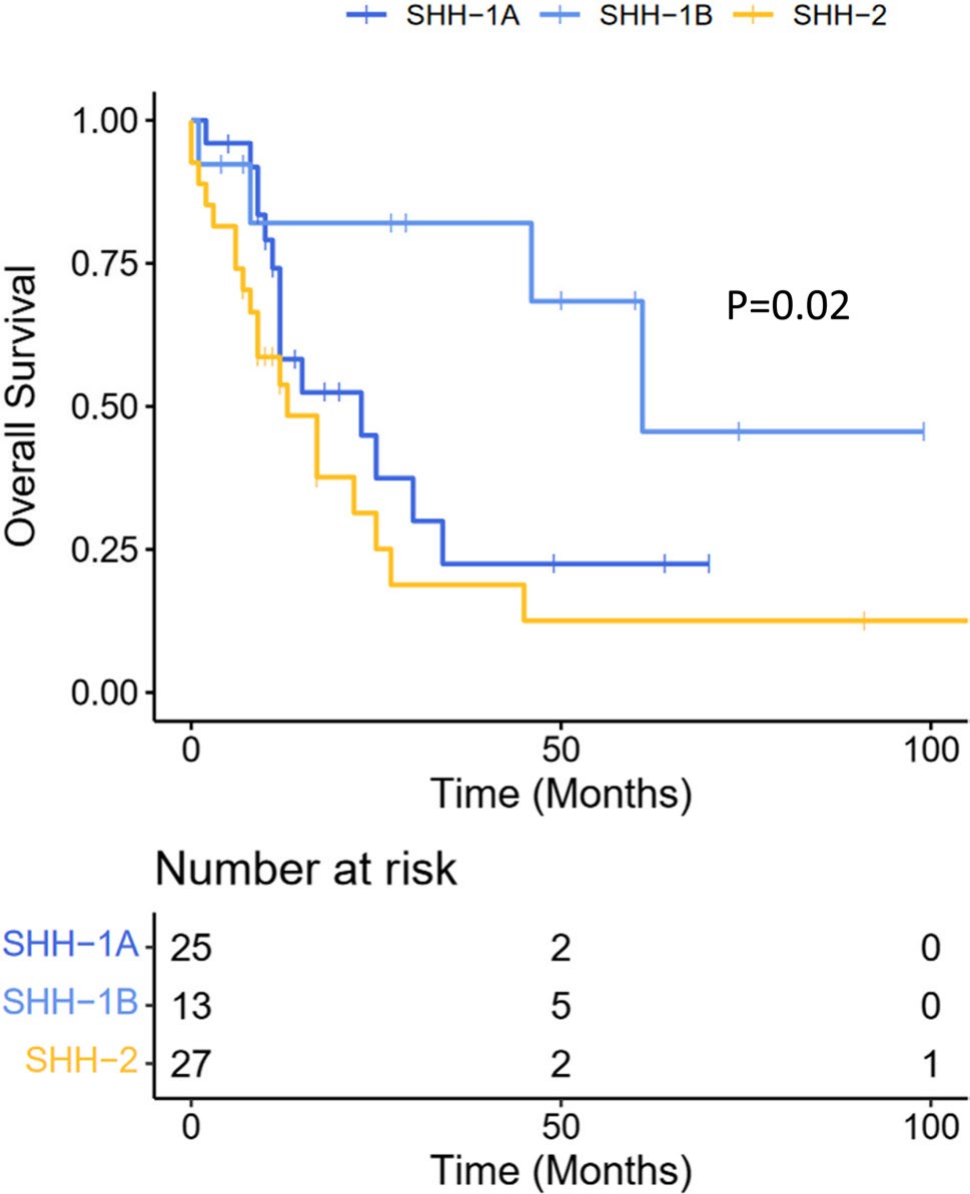
Outcome. Kaplan–Meier estimates of overall survival in ATRT–SHH subgroups SHH-1A, SHH-1B and SHH-2. *P* = 0.02 Log-Rank Test between the three subgroups

On univariate Kaplan–Meier survival analysis, molecular subgroup status had prognostic impact. Patients harboring SHH-1B experienced significantly longer overall survival when compared to SHH-1A and SHH-2 patients (median 61 vs. 23 months and 13 months, respectively; Log-Rank *P* = 0.02; Fig. 7). On univariate analyses, longer overall survival was also associated with older age (> 3 years) and positive ASCL1 staining, but not OLIG2 or GFAP staining, tumor location, gross total resection or the presence of pathogenic/likely pathogenic *SMARCB1* germline variants (Table 1). On multivariate Cox-Regression analysis (Backward Stepwise Wald approach), taking into account molecular subgroup, age category and ASCL1 staining status (i.e., all variables significant at univariate analysis), only age and molecular subgroup remained independently associated with overall survival (Supplementary Table 3). These data suggest that molecular subtyping of ATRT–SHH is of clinical relevance, as patients above 3 years harboring tumors of the SHH-1B subgroup are characterized by relatively favorable outcome. In our series, these cases represented 13/65 (20%) of ATRT–SHH. A total of 9/13 patients (69%) were alive after a median follow-up of 29 months.

**Table 1:** Survival analysis

Factor	*P*
Age (< 3 years vs ≥ 3 years)	0.002
Molecular subgroup (SHH-1A vs. SHH-1B vs. SHH-2)	0.020
ASCL1 staining status (Present vs. focal or present)	0.017
Tumor location (Supratentorial vs. infratentorial vs. both compartments)	0.21
Gross total resection (Achieved vs. not achieved)	0.75
OLIG2 staining status (Present vs. focal or absent)	0.74
GFAP staining status (Present vs. focal or absent)	0.26
Pathogenic/likely pathogenic *SMARCB1* germline variant (Present vs. absent)	0.19

Prognostic role of clinical and molecular factors on overall survival in ATRT–SHH. Univariate analyses using Log-Rank Test

## Discussion

The observation that the largest molecular group of ATRT, ATRT–SHH, comprises distinct subgroups of clinical relevance represents the main finding of the present study. The cohort included SMARCB1-deficient ATRTs that could be unequivocally assigned to the ATRT–SHH molecular group based on DNA methylation-based CNS tumor classification [2], but shows high diversity with regard to patient age, tumor location, tumor volume and histopathological findings, reflecting the high degree of heterogeneity commonly observed in ATRT–SHH. Previous observations [16, 20] already had suggested further separation of ATRT–SHH based on tumor location: ATRT–SHH-1 mostly representing supratentorial tumors and ATRT–SHH-2 associated with infratentorial location. In the present study, we could demonstrate that the heterogeneity within ATRT–SHH is related to three newly defined robust molecular subgroups, which show similarities but also profound differences not only in tumor location, but also with respect to epigenetic landscapes, chromosomal alterations, expression signatures and outcome.

Molecular characterization, in particular DNA methylation profiling, has emerged as an important method for the classification of CNS tumors, as well as a powerful diagnostic tool [2, 3, 32]. In the latest 5th edition of the WHO Classification of CNS tumors [24], many tumors are now defined based on distinct DNA methylation patterns. DNA methylation profiling of our cohort resulted in the identification of three independent and stable clusters: two of them, mainly represented supratentorial tumors and were categorized as SHH-1A and SHH-1B, while the third one, SHH-2, mainly comprised infratentorial tumors, often extending to midline supratentorial structures and the pineal region. Location-dependent heterogeneity based on tumor DNA methylation profiles has been previously described for other CNS tumor entities, such as medulloblastoma [31, 38], ependymoma [6, 30], and pilocytic astrocytoma [23]; however, this particular feature is not always strictly associated with biological and/or clinical relevance. For this reason, we investigated further clinical, histopathological and molecular features across ATRT–SHH subgroups. Patients harboring SHH-1B tumors were remarkably older than SHH-1A and SHH-2 and more often featured chromosomal alterations, whereas SHH-2 represented the youngest patients and a remarkably high proportion of *SMARCB1* germline mutations, which might partly contribute to the well-established association of pathogenic/likely pathogenic *SMARCB1* germline variants and younger age [27].

We also noted different protein expression patterns across the molecular subgroups. Whereas the vast majority of SHH-1B stained positive for proneural marker ASCL1, staining was less frequent in SHH-1A and SHH-2. These findings confirm the notion that ASCL1 is not uniformly expressed in ATRT–SHH [16] and clearly argue against a role of ASCL1 as a surrogate marker [40] for the diagnosis of ATRT–SHH. In contrast, a significant number of supratentorial SHH-1A and SHH-1B cases stained positive for glial markers OLIG2 and GFAP. While expression of GFAP in a proportion of ATRT had already been noted by Rorke et al. [35], OLIG2 expression in ATRT is less well characterized. In a series of 15 ATRT examined by immunohistochemistry, absent OLIG2 expression has been reported [26]. Interestingly, 14/15 cases of that study were of infratentorial location. This is well in line with our notion that OLIG2 expression is virtually absent in SHH-2, which represent the majority of infratentorial ATRT–SHH. The fact that expression of GFAP and OLIG2 was encountered in older patients harboring SHH-1A and SHH-1B represents a diagnostic pitfall that needs to be considered in the differential diagnosis of malignant gliomas in children and young adults. Immunohistochemistry for SMARCB1 as well as DNA methylation profiling will allow for identification of such cases as ATRT–SHH. Even though none of our cases displayed characteristic genetic or epigenetic features of malignant glioma and none showed a component with retained SMARCB1 staining, the possibility that some ATRT–SHH expressing glial markers might rather represent unusual glial neoplasms, in which SMARCB1 deficiency may have caused epigenetic similarity with ATRT–SHH needs to be considered. ASCL1 has a key role in the regulation of neurogenesis and differentiation of neuronal progenitor cells [1, 13]. In this context, it is interesting that co-expression of OLIG2 and ASCL1 has been found to be relevant for the specification of oligodendrocyte progenitors during development [37], while OLIG2+/GFAP+ mature astrocyte populations have been described in the forebrain, cerebral cortex and striatum [25]. Differential expression patterns of ASCL1, OLIG2 and GFAP in ATRT–SHH subgroups could thus reflect distinct functional or cellular identities, whereas ASCL1 expression across subgroups could indicate various degrees of differentiation.

Gene expression profiling of the three ATRT–SHH subgroups provided additional details on their molecular status. SHH-1A and SHH-1B tumors overexpressed genes linked to the development of central nervous system components and/or structures. *GSX1* and *FOXG1* overexpressed in SHH-1A are known to be predominantly expressed in the cerebral cortex and to have a functional role throughout brain development; SHH1-B tumors showed overexpression of genes linked to neuronal specification (*NEUDOD6*) [42] but also *CACNG3,* for which a role in the biology of malignant gliomas has been suggested [44]. In contrast, SHH-2 tumors displayed an enrichment of genes involved in the hindbrain development. In particular, *EN2* in humans has a role in regulating cerebellar and midbrain development [5, 7]. The gene is also expressed in immature dopaminergic neurons [43]. Differentially expressed genes in the three subgroups might reflect tumor origin from different brain regions, but could also imply distinct functional roles or tumorigenic properties that may be driven by different cells of origin.

Importantly, all three ATRT–SHH subgroups showed comparable overexpression of both SHH and Notch pathway members. In contrast to SHH-activated medulloblastoma, activating mutations are generally absent in ATRT–SHH [16], suggesting other mechanisms may be operative. Indeed, knockdown of *SMARCB1* has been shown to cause SHH activation and overexpression of *GLI1* [18]. On the other hand, inhibition of Notch signaling has been shown to affect growth of ATRT–SHH cell lines [39], suggesting that misregulation of SHH signaling alone may not be sufficient for tumorigenesis. In the fly model, knockdown of *Snr1* in hedgehog activated cells altered hedgehog signaling but also caused Notch activation and tumor formation. Notch activation under these conditions could be a direct or indirect result of *Snr1* knockdown*.* Nevertheless, these results further suggest that SHH and Notch signaling are both active and tightly interrelated under conditions of SMARCB1-deficiency. Disturbed cross-talk between SHH and Notch signaling may lead to parallel or non-synchronous pathway activation and tumorigenesis in ATRT–SHH.

Finally, survival analysis also demonstrated significant differences between the three SHH subgroups, with longer overall survival linked to molecular subgroup SHH-1B, age above 3 years and positive ASCL1 staining status. These findings further confirm the important role of patient age, but also highlight a potential clinical role of molecular subgroup status. The fact that ASCL1 expression was enriched in SHH-1B, which mainly comprises supratentorial tumors in older patients, is well in line with previous observations suggesting a better prognosis of supratentorial ASCL1-positive ATRT [40]. Even though in our series only age and molecular subgroup status were independent prognostic markers of overall survival, the potential prognostic role of ASCL1 staining warrants further investigation. The absence of a prognostic role of ATRT–SHH in general observed within the EU-RHAB cohort (from which many cases of the present series were contributed) could well be explained by a higher proportion of SHH-2 cases as compared to other studies. Indeed, in the Children's Oncology Group Trial ACNS0333 which reported a longer event-free survival for children harboring ATRT–SHH [34], the proportion of ATRT showing an infratentorial and supratentorial component (which represents a characteristic feature of SHH-2) accounted for only 7.5% [34]. Our findings clearly support a prognostic impact of molecular stratification that (along with age and ASCL1 staining status) could be employed for risk assessment of ATRT–SHH patients within future clinical trials.

There are also limitations of the study. As in many studies on rare cancers, the cohort represents a relatively small number of cases and neuroradiological imaging data or material for ancillary immunohistochemical studies could not be retrieved for all cases. In addition, sample collection and analysis were conducted in retrospect and not prospectively within a controlled clinical trial. Even though the majority of patients had been treated according to EU-RHAB recommendations, the size of the cohort was certainly not large enough to control for the effect of various chemotherapy protocols and radiotherapy. Finally, follow-up was relatively short. Future studies aiming to prospectively examine a larger number of cases to consolidate and expand our findings are desirable.

In conclusion, ATRT–SHH comprises three subgroups characterized by SHH and Notch pathway activation, but divergent molecular and clinical features. Our data suggest that molecular subgrouping of ATRT–SHH has prognostic relevance and might aid to stratify patients within future clinical trials.

## Supplementary Information

Below is the link to the electronic supplementary material.

Supplementary file1 (PDF 1012 KB)

Supplementary file2 (XLSX 30 KB)

Supplementary file3 (XLSX 170 KB)

Supplementary file4 (PDF 39 KB)
