# Supplementary material for: ATRT–SHH comprises three molecular subgroups with characteristic clinical and histopathological features and prognostic significance
Source: Acta Neuropathol. 2022 Apr 30;143(6):697–711. doi: 10.1007/s00401-022-02424-5 (PMC9107423; doi:10.1007/s00401-022-02424-5)
Supplement: Supplementary file 1 — Supplementary file1 (PDF 1012 KB) [file 401_2022_2424_MOESM1_ESM.pdf]

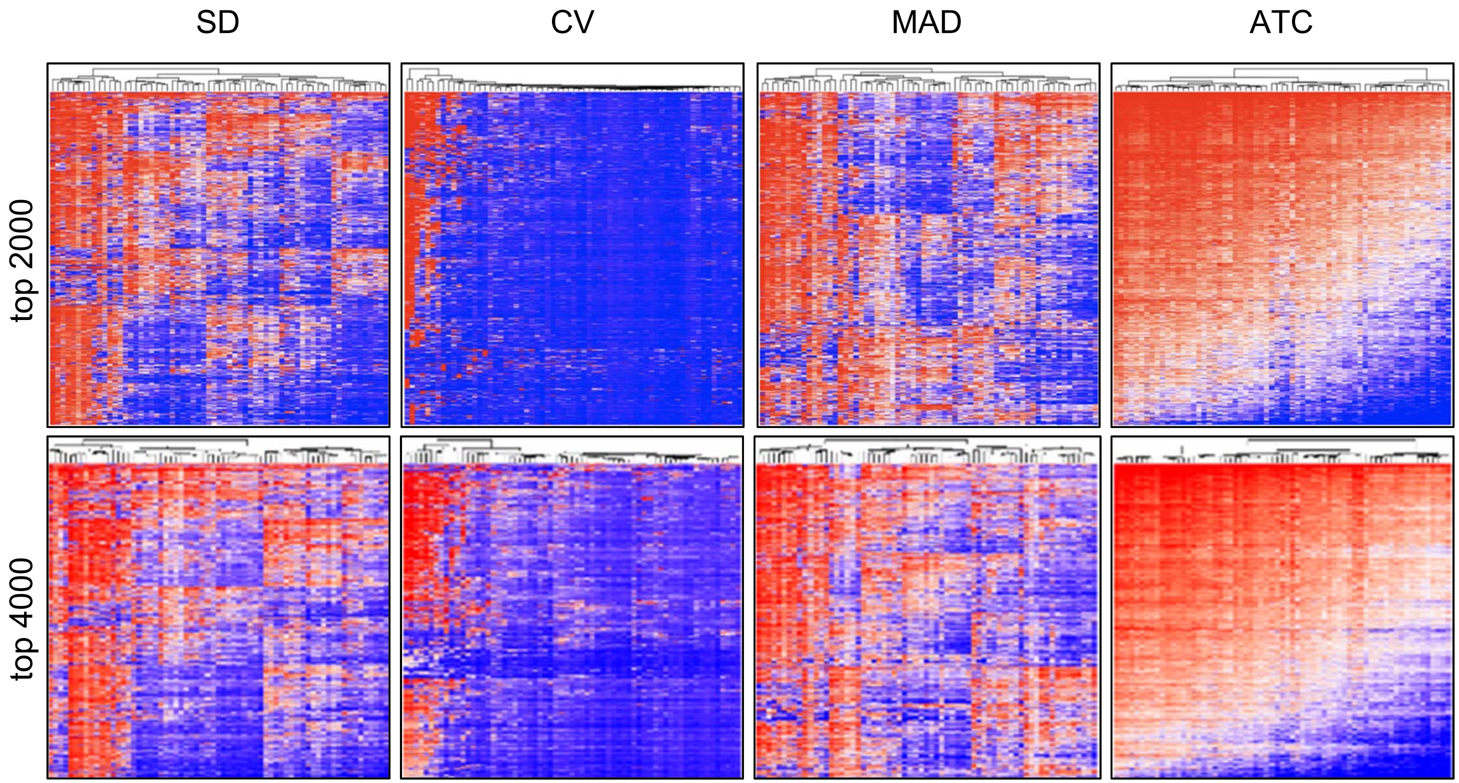

Supplementary Figure S1: Comparison of different feature selection methods. Methylation profiles using the top 2000 (top row) and top 4000 (bottom row) CpG probes extracted with different feature selection methods (SD = standard deviation, CV coefficient of variance, MAD = median absolute deviation, ATC = ability to correlate to other rows). CpG probes selected by SD show the most distinctive methylation profile across samples, while MAD selects probes which generate a more noisy profile. CV tends to extract probes with generally lower methylation values and less distinctive patterns. Probes selected by ATC show similar patterns across sample with merely gradual methylation changes.

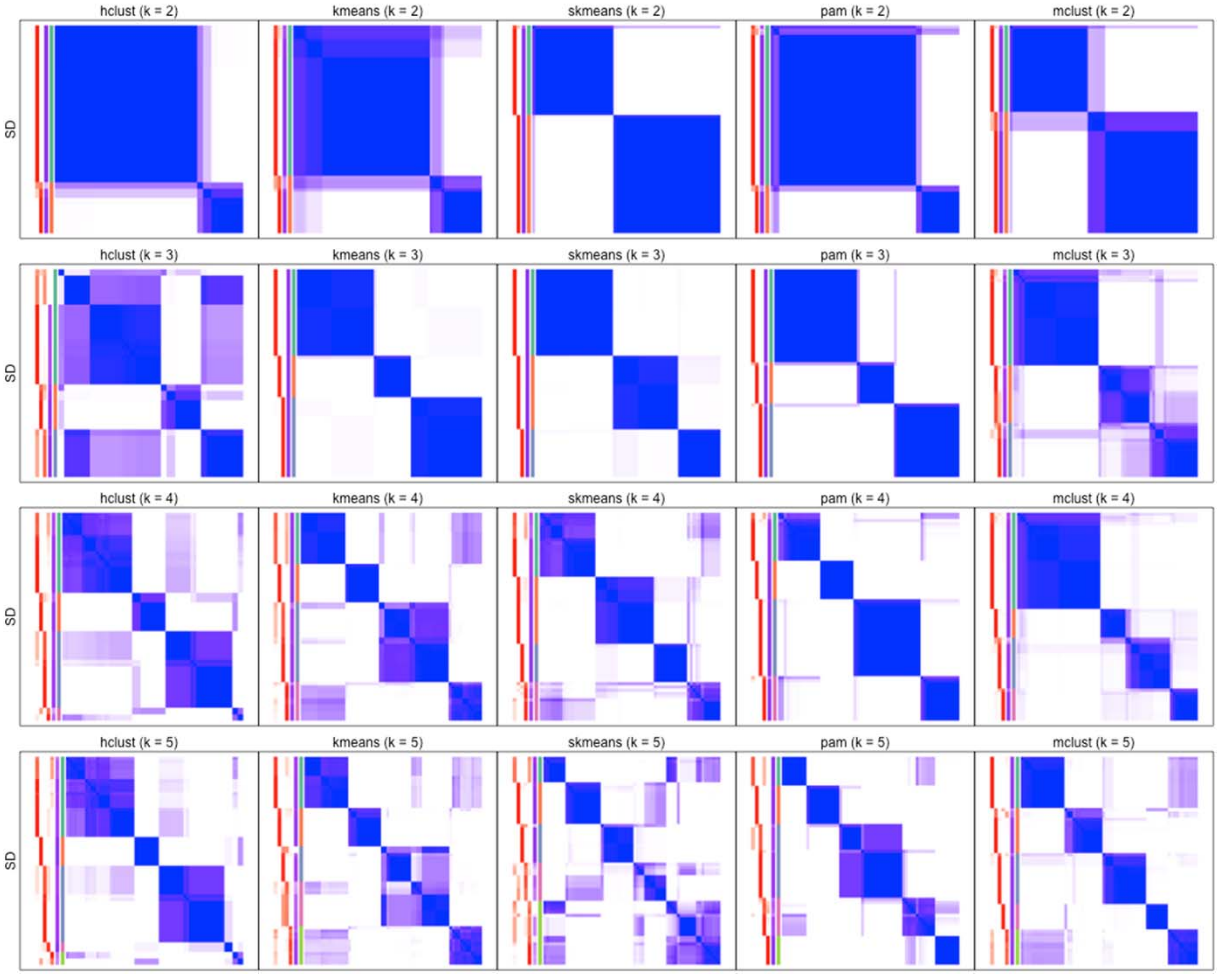

Supplementary Figure S2: Consensus matrices for different partitioning methods. Consensus heatmaps for different clustering algorithms across iterations of k clusters. Most stable partitioning is observed for k=3 using k-means clustering (second row, second item).

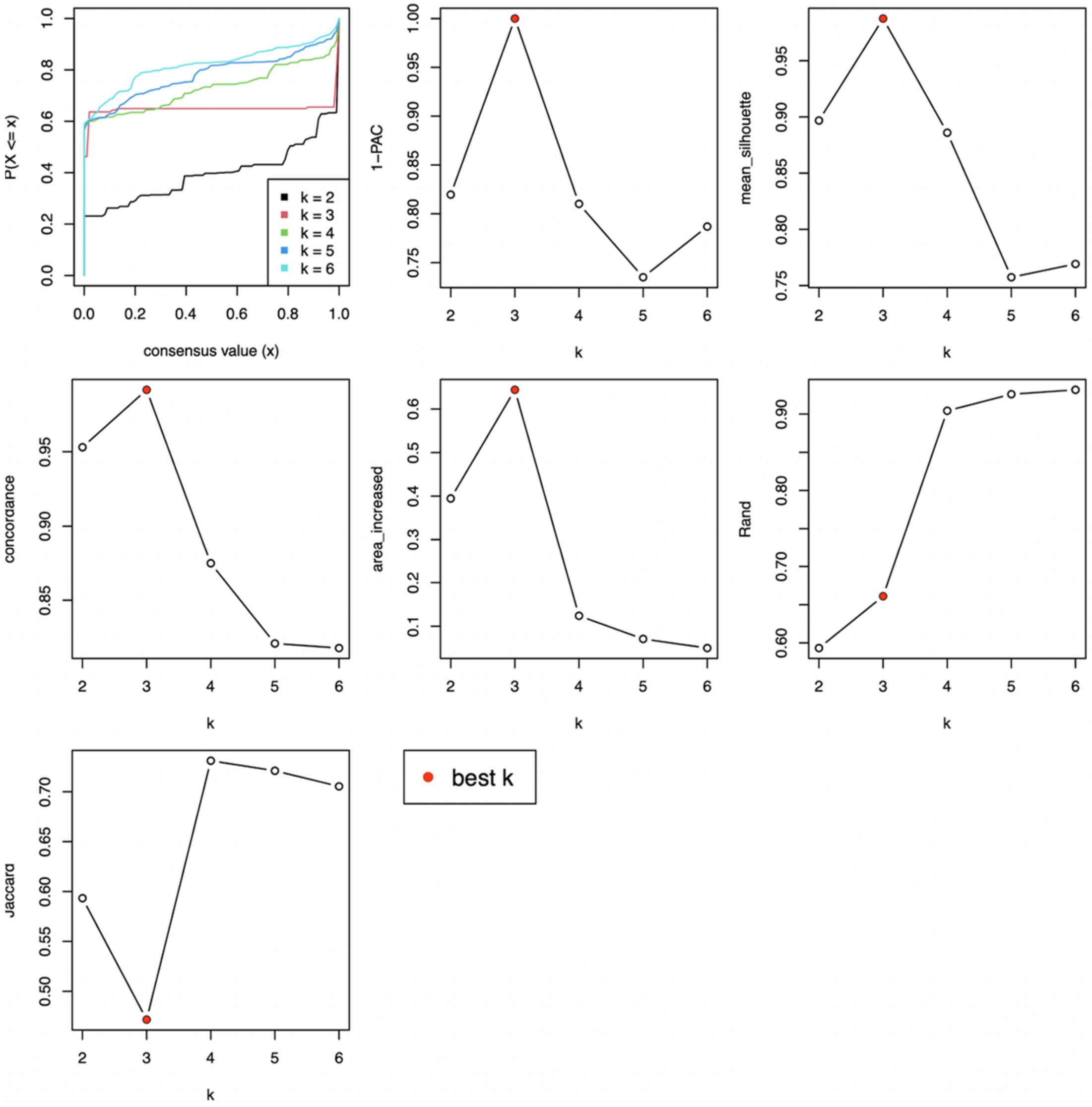

Supplementary Figure S3: Optimal number of k clusters. eCDF curves, 1-PAC (proportion of ambiguous clustering), mean silhouette scores, concordance (mean probability of fiting the consensus subgroup labels in all partitions), area increased (referring to the eCDF curves), Rand index (percent of pairs of samples that are both in the same cluster or both not in the same cluster) and Jaccard index (ratio of pairs of samples are both in the same cluster in the partition of k and k-1) suggest k=3 as the optimal number of clusters when SD is set as the feature selection method and k-means clustering is applied.

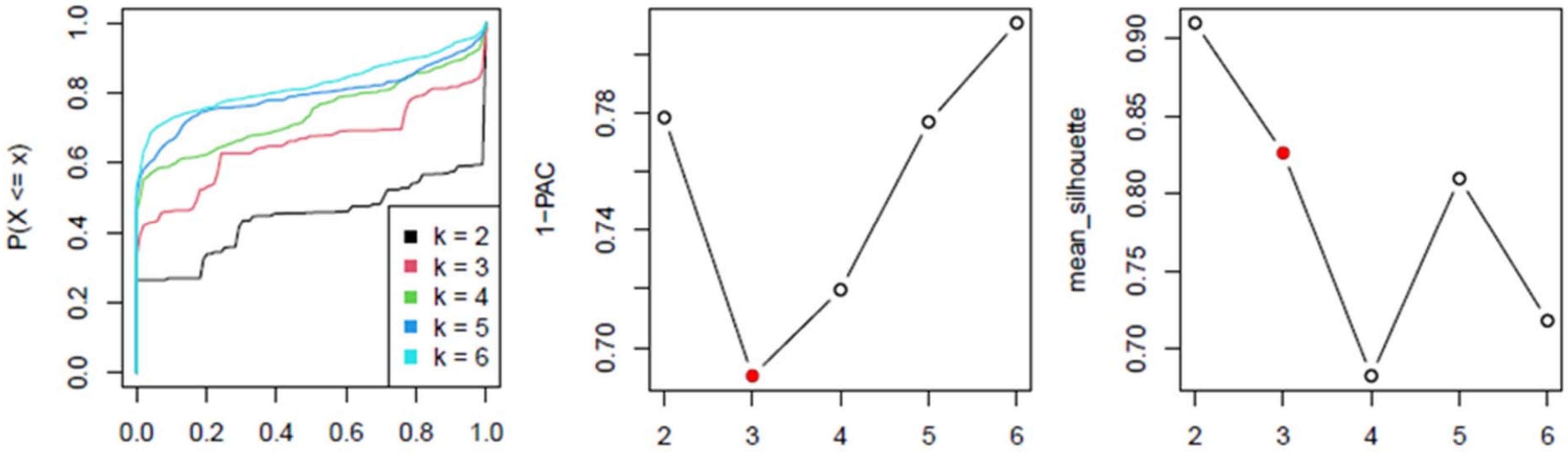

Supplementary Figure S4: Consensus cluster analysis of a larger ATRT-SHH cohort. cola subclustering analysis of study cases (n=65) and additional ATRT-SHH samples with available gene expression data (n=22) confirmed k=3 as most stable clusters. Additional tumor samples were then assigned to the matching ATRT-SHH subgroup.

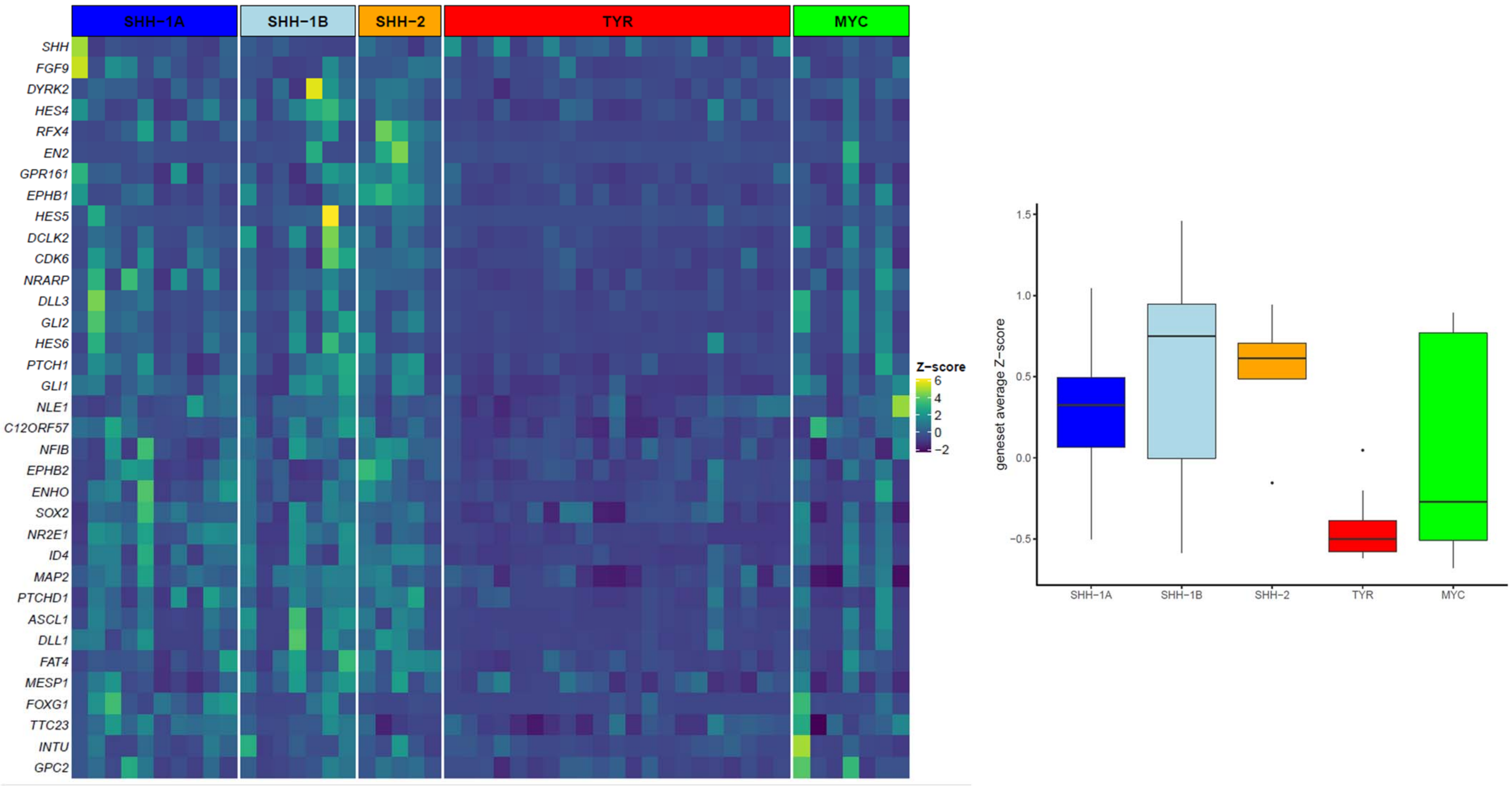

Supplementary Figure S5: Expression of genes coding for members of the SHH and Notch pathway across ATRT-SHH subgroups as compared to ATRT-TYR and ATRT-MYC. Heatmap representing the expression levels (Z-score) of genes coding for members of the SHH and Notch pathway in ATRT-SHH subgroups as compared to ATRT-TYR and ATRT-MYC as well as boxplot showing the average Z-score values over genes coding for members of the SHH and Notch pathway.
