# Supplementary material for: ATRT–SHH comprises three molecular subgroups with characteristic clinical and histopathological features and prognostic significance
Source: Acta Neuropathol. 2022 Apr 30;143(6):697–711. doi: 10.1007/s00401-022-02424-5 (PMC9107423; doi:10.1007/s00401-022-02424-5)
Supplement: Supplementary file 4 — Supplementary file4 (PDF 39 KB) [file 401_2022_2424_MOESM4_ESM.pdf]

Supplemental Table 3: Multivariate Analysis (Cox Regression model).

Variable		B	SE	Wald	P	Exp(B)
Step 1	Molecular Subgroup	.356	.193	3.405	.065	1.427
	Age	-1.494	.629	5.632	.018	.225
	ASCL1	-.249	.206	1.468	.226	.779
Step 2	Molecular Subgroup	.311	.189	2.712	.100	1.365
	Age	-1.617	.621	6.785	.009	.199

		Change previous step			Change previous block		
Step	-2 Log-Likelihood	Chi-square	df	P	Chi-square	df	P
1	199.316	12.245	3	.007	13.046	3	.005
2	200.800	10.684	2	.005	1.485	1	.223
